# Biological characterization of purified macrophage-derived neutrophil chemotactic factor

**DOI:** 10.1155/S0962935195000421

**Published:** 1995-07

**Authors:** M. Dias-Baruffi, M. C. Roque-Barreira, F. Q. Cunha, S. H. Ferreira

**Affiliations:** 1 Department of Immunology Faculty of Medicine Ribeirão Preto São Paulo, 14 049-900 Brazil; 2 Department of pharmacology Faculty of Medicine Ribeirão Preto São Paulo, 14 049-900 Brazil

## Abstract

We have recently described the purification of a 54 kDa acidic protein, identified as macrophage-derived neutrophil chemotactic factor (MNCF). This protein causes *in vitro* chemotaxis as well as *in vivo* neutrophil migration even in animals treated with dexamethasone. This *in vivo* chemotactic activity of MNCF in animals pretreated with dexamethasone is an uncommon characteristic which discriminates MNCF from known chemotactic cytokines. MNCF is released in the supernatant by macrophage monolayers stimulated with lipopolysaccharide (LPS). In the present study, we describe some biological characteristics of homogenous purified MNCF. When assayed *in vitro*, MNCF gave a bell-shaped dose–response curve. This *in vitro* activity was shown to be caused by haptotaxis. Unlike N-formyl-methionylleucyl- phenylalanine (FMLP) or interleukin 8 (IL-8), the chemotactic activity of MNCF *in vivo* and *in vitro*, was inhibited by preincubation with D-galactose but not with D-mannose. In contrast with IL-8, MNCF did not bind to heparin and antiserum against IL-8 was ineffective in inhibiting its chemotactic activity. These data indicate that MNCF induces neutrophil migration through a carbohydrate recognition property, but by a mechanism different from that of the known chemokines. It is suggested that MNCF may be an important mediator in the recruitment of neutrophils via the formation of a substrate bound chemotactic gradient (haptotaxis) in the inflamed tissues.


Mediators of Inflammation 4, 263–269 (1995)

### Biological characterization of purified macrophage-derived neutrophil chemotactic factor

M. Dias-Baruffi,[1] M. C. Roque-Barreira,[1,CA] F. Q. Cunha[2] and S. H. Ferreira[2]

Departments of [1]Immunology and [2]Pharmacology, Faculty of Medicine, Ribeirão Preto, São Paulo, 14,049-900, Brazil

[CA]Corresponding Author

**We have recently described the purification of a 54 kDa acidic protein, identified as macrophage-derived neutrophil chemotactic factor (MNCF). This protein causes *in vitro* chemotaxis as well as *in vivo* neutrophil migration even in animals treated with dexamethasone. This *in vivo* chemotactic activity of MNCF in animals pretreated with dexamethasone is an uncommon characteristic which discriminates MNCF from known chemotactic cytokines. MNCF is released in the supernatant by macrophage monolayers stimulated with lipopolysaccharide (LPS). In the present study, we describe some biological characteristics of homogenous purified MNCF. When assayed *in vitro*, MNCF gave a bell-shaped dose–response curve. This *in vitro* activity was shown to be caused by haptotaxis. Unlike *N*-formyl-methionyl-leucyl-phenylalanine (FMLP) or interleukin 8 (IL-8), the chemotactic activity of MNCF *in vivo* and *in vitro*, was inhibited by preincubation with D-galactose but not with D-mannose. In contrast with IL-8, MNCF did not bind to heparin and antiserum against IL-8 was ineffective in inhibiting its chemotactic activity. These data indicate that MNCF induces neutrophil migration through a carbohydrate recognition property, but by a mechanism different from that of the known chemokines. It is suggested that MNCF may be an important mediator in the recruitment of neutrophils via the formation of a substrate bound chemotactic gradient (haptotaxis) in the inflamed tissues.**

**Key words:** *Chemotaxis, Cytokine, Glucocorticoids, Inflammation, Lectins, Leukocyte*

### Introduction

Emigration of neutrophils from the circulating blood to the site of the injury is a cardinal event during the inflammatory process. This phenomenon is complex and mediated by various molecules acting in different ways.[1] Interleukin 1 and 8 (IL-1, IL-8), tumour necrosis factor (TNF), and the complement fragments and leukotriene $B_4$ ($LTB_4$) are macrophage products thought to be involved in the migration of neutrophils to the inflamed site, a phenomenon characteristic of the acute phase of the inflammatory process.[2–5] Some products such as C5a and $LTB_4$ may have a direct chemotactic effect on neutrophils.[6–8] Others may act indirectly by mechanisms which depend on the tissue's resident cells and on the endothelium. For example, in addition to their effects on neutrophils and endothelial cells, where they increase the expression of adhesion molecules,[9–13] IL-1 and TNF also stimulate the release of chemotactic factors by resident macrophages.[11] In fact, the neutrophil-induced migration into the peritoneal cavity by cytokines such as IL-1 and TNF, as well as various inflammatory stimuli (LPS, carrageenin, etc), is dependent on the number of resident cells. This idea is supported by the alteration in the magnitude of the neutrophil migration induced by various stimuli when the resident cell population in the peritoneum is increased by previous treatment with thioglycollate or reduced by cell depletion.[11,14–17]

Glucocorticoids greatly reduce the neutrophil migration induced by inflammatory stimuli or by cell-dependent chemotactic cytokines. The mechanism by which glucocorticoids inhibit cell migration is still controversial. There are some indications that these substances block the expression of adhesion molecules in the endothelium.[18] However, there is also evidence that glucocorticoids may not have a direct effect on the expression of these proteins in neutrophils or endothelial cells but instead may block the

© 1995 Rapid Science Publishers release of the mediators involved in the final stage of neutrophil emigration into tissue.[19,20]

In the companion paper[21] we describe the isolation of a homogeneous protein which, like crude MNCF, induced neutrophil migration *in vitro* as well as *in vivo* even in animals pretreated with dexamethasone. The purified factor was acidic and had a molecular mass of 54 kDa. In the present study, we describe some biological characteristics of this purified homogeneous MNCF fraction associated with their neutrophil chemoattractant activity *in vivo* and *in vitro*. These activities are dependent upon the carbohydrate recognition property of MNCF.

### Materials and Methods

*Animals:* Male, albino, Wistar rats (*Rattus norvegicus*) weighing from 180 to 200 g and maintained in temperature-controlled rooms at 23–25°C with free access to food and water, were used as the source of peritoneal macrophages as well as for the *in vivo* tests of cell migration.

*Production and purification of neutrophil chemotactic factor (MNCF):* MNCF was produced and purified as described by Cunha and Ferreira, and Dias-Baruffi *et al.*[14,22] Briefly, the supernatant from LPS-stimulated macrophage monolayers (crude MNCF) were submitted to a simple two-step purification process involving adsorption to a D-galactose column followed by gel filtration on Superdex 75, for the isolation of MNCF. In the present paper MNCF refers to the homogeneous fraction obtained after the last purification step.

*Heparin binding assay of MNCF:* Binding to heparin was assessed with a heparin–agarose column. The column was washed and equilibrated with PBS. Three ml of crude MNCF derived from $4.2 \times 10^8$ cells were applied to a 4 ml column of heparin–agarose (Sigma Chemical Company, St. Louis, MO, USA) at 4°C. The material not retained by the column after washing with PBS was denoted as the heparin-not-bound fraction while that retained and eluted with 0.5 M NaCl in PBS was denoted heparin-bound. Both fractions were applied to a Fast Desalting HR 10/10 column (Pharmacia LKB Biotechnology, Uppsala, Sweden). The column was developed with sterile deionized water at 20°C and a flow rate of 0.5 ml/min. The eluate absorbance was monitored at 206 and 280 nm, and fractions of 0.5 ml were collected. These fractions were then tested either *in vivo* for their ability to induce neutrophil migration into the peritoneal cavity of rats pretreated or not with glucocorticoids, or *in vitro* for their ability to induce neutrophil migration in a microchamber in the presence of either anti-IL-8 serum or of control serum.

*Determination of biological activity:*

In vivo *migration assays.* MNCF activity was assayed by its ability to induce neutrophil migration into the peritoneal cavity or air pouch of rats pretreated with a glucocorticoid (0.5 mg of dexamethasone acetate ester/kg, subcutaneously; Merck, Darmstadt, Germany). Each sample was tested in groups of five to six rats. Air pouches were produced on the dorsum of rats as described by Edwards *et al.*[23] One h after dexamethasone, 1 or 3 ml samples were injected into the air pouch or peritoneal cavity, respectively. The animals were sacrificed by cervical dislocation 4 or 6 h later for the peritoneal or air pouch tests, respectively. The cells were immediately harvested by washing the respective cavities with 5 or 10 ml PBS containing albumin (0.1% w/v) and heparin (5 IU/ml). Total counts of harvested cells were performed in a Neubauer chamber. Differential counts were made on smears stained using Rosenfeld's panchromic method. The results are reported as the mean number (± S.E.M.) of neutrophils per ml of cavity wash.

To test the inhibitory effect of D-galactose or D-mannose on the neutrophil migration induced by MNCF or other stimuli, the samples were incubated with these sugars (final concentration 0.4 M) for 30 min at room temperature. The neutrophil migration activity of the stimuli was then tested in the air pouch model as described above.

In vitro *neutrophil chemotaxis migration assay.* Assays of *in vitro* neutrophil migration were performed as described by Bignold[24] in a 48-well chemotactic microchamber (Neuroprobe, Cabin John, MD, USA). Human neutrophils were isolated from the heparinized peripheral blood of healthy human volunteers using mono poly resolving medium (Flow laboratories, Rockville, MD, USA). After washing with RPMI 1640 medium, the neutrophils were resuspended in RPMI 1640 medium containing 0.1% (w/v) BSA (RPMI–BSA), to provide $10^6$ cells/ml. Typical preparations contained more than 95% viable neutrophils. Purified neutrophils were placed in the upper chamber while the lower chamber contained the test samples dissolved in RPMI–BSA. Random migration was assessed by using RPMI–BSA in the lower chamber. The peptide FMLP ($10^{-7}$ M) was used as the reference chemoattractant. The number of cells that migrated through the entire thickness of a 5 μm polycarbonate filter (Millipore Corp., Bedford, MA, USA) during the 1 h incubation at 37°C in a 5% $CO_2$ atmosphere was counted. Five fields were counted for each assay and each sample was assayed in triplicate. The results are reported as the mean number (± S.E.M.) of neutrophils per field. The samples tested were crude MNCF, purified MNCF or human recombinant interleukin-8 (rhIL-8; from National Institute for Biological Standards and Control-NIBSC) as well as the heparin-bound and heparin-not-bound fractions of the LPS-stimulated macrophage supernatant. Crude MNCF, purified MNCF or rhIL-8 were incubated in medium with D-galactose or D-mannose (0.4 M in RPMI–BSA) for 30 min at room temperature before testing the effect of D-galactose or D-mannose on the chemotactic activity of each sample. The heparin-bound and heparin-not-bound fractions, as well as rhIL-8, were incubated separately in medium containing anti-IL-8 goat serum or control goat serum (final dilutions = 1:100 and 1:10 in RPMI-BSA) for 30 min at room temperature before testing the effect of anti-IL-8 on the chemotactic activity of each sample.

*In vitro neutrophil haptotaxis assay.* The haptotaxis assay was conducted as described by Rot[25] in a 48-well chemotactic chamber (Neuroprobe) by forming a gradient across a 5 μm polycarbonate filter (Millipore Corp.) Positive haptotactic gradients of MNCF were preformed by filling some of the bottom wells with purified MNCF derived from $3.6 \times 10^7$ cells and the corresponding top wells with RPMI–BSA. Negative haptotactic gradients were obtained by reversing the above procedure, i.e., by filling a set of top wells with MNCF derived from $3.6 \times 10^7$ cells and the corresponding bottom wells with RPMI–BSA. When chemotaxis, chemokinesis or random migration were examined, both the upper and lower wells were filled with RPMI–BSA. After incubation at 37°C for 20 min, the chambers were disassembled. The filter was thoroughly washed in an RPMI–BSA bath to remove the nonbound attractant, after which it was air dried and placed in another chemotactic chamber. In this second chamber, RPMI–BSA was used to fill the bottom wells corresponding to those wells with preformed positive or negative gradients in the first chamber. The wells corresponding to those that did not contain attractant in the first chamber were used to assess chemotaxis (by adding the attractants MNCF or FMLP ($10^{-7}$ M) to the bottom chamber), chemokinesis (by adding equal quantities of MNCF to both the top and bottom chambers), or random migration (by adding RPMI–BSA to both the top and bottom chambers). Fifty μl of a suspension of $10^6$ neutrophils/ml were placed in each top well of the second chamber, and their migration was measured by counting the cells that migrated into or towards the second chamber across the filter during a 30 min incubation at 37°C. Five fields of filters were counted for each assay and each sample was assayed at least in triplicate. The results are reported as mean ± S.E.M.

### Results

*Biological characterization of chemotactic activities of MNCF:* Purified MNCF induced neutrophil migration in the same dose-dependent manner in both normal and dexamethasone-pretreated rats (Fig. 1A). The dose–response curve obtained for the *in vitro* chemotactic activity of purified MNCF was bell-shaped (Fig. 1B). The chemotactic activity was shown to result from haptotaxis rather than chemokinesis since the neutrophil chemokinetic migration induced by purified MNCF was similar to that of a negative control. While *in vitro* neutrophil migration was observed with a positive haptotactic gradient with MNCF, the negative haptotactic gradient of MNCF was unable to induce neutrophil migration (Fig. 2). These data indicate that the neutrophil migration induced by filter-bound MNCF was gradient-driven.

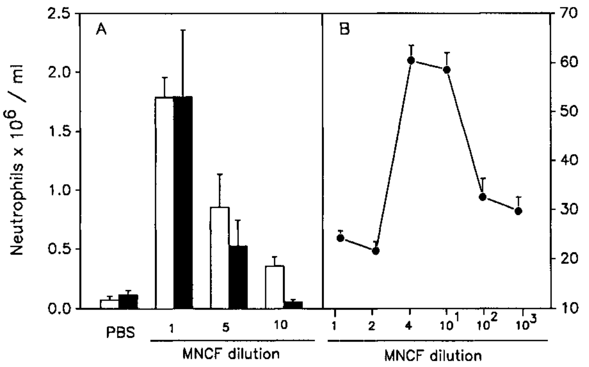

FIG. 1. Dose-response curves for the *in vivo* and *in vitro* neutrophil migration induced by purified MNCF. Panel A: Neutrophil migration into the peritoneal cavity of normal (open bars) or dexamethasone-pretreated rats (0.5 mg/kg; filled bars) induced by purified MNCF at the dilutions indicated. The quantity of MNCF injected per rat at 1:1 dilution was obtained from $1.4 \times 10^7$ macrophages. PBS was also injected. Neutrophil migration was evaluated 4 h after the injection of MNCF. The results are the mean number (± S.E.M.) of neutrophils per ml of peritoneal wash ($n$ = six rats/group). Panel B: Neutrophil chemotaxis *in vitro* induced by purified MNCF at the dilutions indicated. The quantity of MNCF used in the initial dilution was obtained from $3.6 \times 10^7$ macrophages. The number of purified human neutrophils used was $10^6$ cells/well. The assay was carried out in triplicate. Migration was evaluated after 1 h. The results are the mean number (± S.E.M.) of neutrophils per field (15 fields).

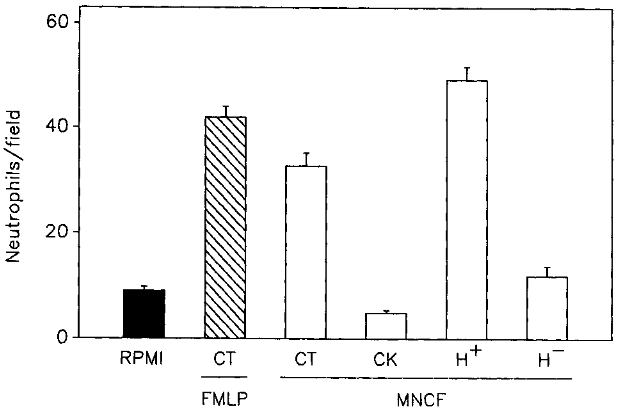

FIG. 2. Purified MNCF induces neutrophil migration *in vitro* by chemotactic and haptotactic gradients. The chemotactic activity of purified MNCF (from 1.4 x $10^7$ macrophages/well) was tested in a 48-well microchamber. The *in vitro* chemotactic (CT), chemokinetic (CK), positive haptotactic ($H^+$) and negative haptotactic ($H^-$) activities were determined as described in the Materials and Methods. The RPMI 1640 medium and FMLP ($10^{-7}$M) were used as negative and positive controls, respectively. The number of purified human neutrophils used was $10^6$ cells/well and, the assay was carried out in triplicate. The results are the mean number ($\pm$ S.E.M.) of neutrophils per field (15 fields).

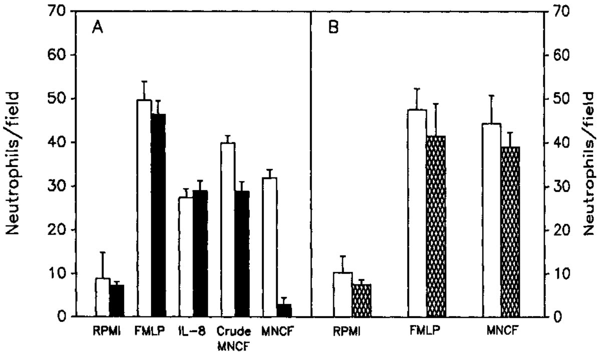

FIG. 3. The effect of D-galactose and D-mannose on the *in vitro* neutrophil migration induced by different stimuli. FMLP ($10^{-7}$M), IL-8 ($10^{-7}$M), crude MNCF (from 2 x $10^6$ macrophages), and purified MNCF (from 1.4 x $10^7$ macrophages) were incubated with RPMI 1640 (open bars) or with D-galactose (D-gal; filled bars; panel A) or D-mannose (D-man; cross hatched bars; panel B) at a concentration of 0.4 M for 30 min and the resulting chemotactic activity was evaluated in a 48-well microchamber. RPMI 1640 either D-gal or D-man were used as negative controls. The number of purified human neutrophils used was $10^6$ cells/well and the assay was carried out in triplicate. Migration was evaluated after 1 h. The results are the mean number ($\pm$ S.E.M.) of neutrophils per field (15 fields).

Table 1 shows that pretreating the test animals with dexamethasone blocked the neutrophil migration induced by the heparin-bound fraction and by rhIL-8. However, as with the results obtained for purified MNCF (Fig. 1), this treatment did not affect the neutrophil migration induced by the heparin-not-bound fraction. In the *in vitro* assay, the chemotactic activity of the heparin-bound fraction was inhibited by preincubation with anti-IL-8 serum. A similar result was observed with rhIL-8. On the other hand, the *in vitro* chemotactic activity of the heparin-not-bound fraction was not affected by the same amount of anti-IL-8 serum.

*Relationship between migration-inducing and carbohydrate-binding activities:* Fig. 3A shows that 0.4 M D-galactose did not affect the chemotactic activity of FMLP or IL-8 *in vitro*, but did inhibit the activity of purified MNCF by more than 90%. As expected, the chemotactic activity of crude MNCF was only partially reduced (28%) by the same concentration of D-galactose. At the same concentration, a non-specific sugar (D-mannose) had no effect on the chemotactic activities of FMLP or purified MNCF (Fig. 3B).

The incubation of crude or purified MNCF with D-galactose (0.4 M) also inhibited the ability to induce neutrophil migration into the air pouch

Table 1. The effect of dexamethasone or anti-IL-8 serum on the chemotactic activities of heparin-bound and heparin-not-bound fractions of LPS-stimulated macrophage supernatants

| | *In vivo* assay[a] | | | *In vitro* assay[b] | | |
|---|---|---|---|---|---|---|
| | Neutrophils x $10^6$ | | Inhibition (%) | Neutrophils/field | | Inhibition (%) |
| | PBS pretreatment | Dexamethasone pretreatment | | Control serum | Anti-IL-8 serum | |
| Medium | 0.2 ± 0.1 | 0.2 ± 0.1 | 0 | 182.0 | 181.9 | 0 |
| rhIL-8 | 2.4 ± 0.4 | 0.4 ± 0.1 | 83 | 603.3 | 201.4 | 67 |
| Heparin-bound | 3.1 ± 0.1 | 1.1 ± 0.1 | 64 | 754.1 | 443.9 | 42 |
| Heparin-not-bound | 1.8 ± 0.1 | 1.8 ± 0.3 | 0 | 604.8 | 664.2 | 0 |

[a] *In vivo* assay: the migration of neutrophils into the peritoneal cavity of rats, pretreated or not with dexamethasone (0.5 mg/kg/s.c., 1 h previously) in response to human recombinant interleukin 8 (rhIL-8; 7.5 moles) or heparin-bound or heparin-not-bound fractions of LPS-stimulated macrophage supernatants (derived from 1.4 x $10^7$ cells). The number of neutrophils is reported as the mean number S.E.M. of cells per ml of peritoneal wash for five animals. PBS was used as the medium control.

[b] *In vitro* assay: the chemotactic activities of rhIL-8 (30 nM) or heparin-bound or heparin-not-bound fractions of LPS-stimulated macrophage supernatants (derived from 4.5 x $10^6$ cells per well) preincubated with control goat serum (1:10) or anti-IL-8 goat serum (1:10) were assayed as described in the Materials and Methods. A 1:100 dilution of both sera was ineffective. The data are reported as the mean S.E.M. of three determinations. RPMI was used as the medium control.

Table 2. Effect of D-galactose and D-mannose on migration *in vivo* induced by MNCF

| | *In vivo* assay | |
|---|---|---|
| | Inhibition (%) | |
| | By D-mannose | By D-galactose |
| Medium | 6 | 0 |
| Crude MNCF | 0 | 40 |
| Purified MNCF | 11 | 46 |

Crude MNCF (from 3 × $10^6$ macrophages), or purified MNCF (from 7 × $10^6$ macrophages) previously non-incubated or incubated with D-galactose or D-mannose (final concentration 0.4 M), was injected into the air pouches of dexamethasone-pretreated rats (0.5 mg/kg). Neutrophil migration was evaluated 4 h later. The results represent the percentage inhibition compared with the migration induced by the same stimulus when non-incubated with D-mannose or D-galactose and incubated with each sugar.

of dexamethasone-pretreated rats by 40 and 46%, respectively. At the same concentration, a non-specific sugar (D-mannose) had no effect on the *in vivo* or *in vitro* chemotactic activity of crude or purified MNCF (Table 2).

### Discussion

In a companion paper,[21] we have described the purification to homogeneity of a 54 kDa acidic protein extracted from the supernatant of LPS-stimulated macrophage monolayers. Like the crude macrophage-derived neutrophil chemotactic factor (MNCF), this protein was able to stimulate neutrophil migration in animals pretreated with dexamethasone.[14,21] This *in vivo* chemotactic activity is an uncommon cytokine characteristic and seems peculiar to MNCF. MNCF behaves like a lectin on the basis of its ability to bind to immobilized D-galactose and the inhibition of its biological activity by the same sugar.[22]

In line with our previous observations for crude MNCF, the isolated protein induced dose-dependent neutrophil migration *in vivo* in naive and as well as in dexamethasone-pretreated animals. D-galactose, but not D-mannose, significantly inhibited (46%) the capacity of purified MNCF to induce neutrophil migration *in vivo*. This limited inhibitory effect of D-galactose in the *in vivo* assay may reflect the diffusion and/or the reabsorption of the sugar from the air pouch cavity during the 6 h required for the neutrophil migration test.

MNCF also caused chemotaxis *in vitro* with the dose-response being similarly bell-shaped to that described for other chemotactic agents such as FMLP and $LTB_4$,[26] D-mannose-binding lectin from *Artocarpus integrifolia* ($KM^+$)[27] and IL-8.[25] The decreased activity observed with high doses of the factor may result from its diffusion into the upper compartment of the chamber, thereby abolishing the chemoattractant gradient. The *in vitro* chemotactic activity of MNCF was strongly inhibited by pre-incubation with D-galactose. This inhibition appears to be specific for MNCF since pre-incubation of FMLP or IL-8 with the same concentration of D-galactose did not affect their chemotactic activity. Pre-incubation with a non-related sugar, D-mannose, did not modify the activity of MNCF or FMLP. The crude MNCF was only partially inhibited by D-galactose since other chemotactic mediators such as IL-1, TNF and IL-8[5,28] were also present. These results support the suggestion that MNCF activity *in vitro* and *in vivo* is associated with a sugar-binding domain of the purified protein.

There are at least three distinct stages in the process of neutrophil migration into perivascular tissues: (1) neutrophil adhesion to the endothelial cells mediated by endothelium-expressed selectins and ICAMs,[29,30]; (2) PECAM-1-mediated neutrophil penetration through endothelial cell junctions;[31,32] and (3) neutrophil migration across the basal membrane into perivascular tissues.[19,20] Haptotaxis is thought to be an important mechanism throughout this whole process. Binding of the chemoattractant on the subendothelial matrix is required for the formation of a haptotactic gradient.[33] In the present paper, we have shown that MNCF causes *in vitro* chemotaxis by both chemotactic and haptotactic mechanisms, properties shared by IL-8.[25] However, in contrast to IL-8, MNCF did not bind to heparin and its chemotactic activity was blocked by D-galactose but not by anti-IL-8 serum (Table 1). Thus, the sugar binding moiety which is involved in the stimulation of neutrophil chemotaxis by MNCF is different from that involved in IL-8-induced chemotaxis.

Early *in vivo* experiments suggested that the inhibitory effect of glucocorticoids on neutrophil migration was due to an action on the neutrophil-endothelium adhesion step.[34–36] There are, however, conflicting results regarding the inhibitory effect of glucocorticoids on the foregoing interaction *in vitro*[37] and on the expression of E-selectin and ICAM-1.[18] Recent *in vivo* monitoring of the different stages of neutrophil migration has shown that the rolling on or adhesion to venular endothelium induced by $LTB_4$ or FMLP was not inhibited by pretreating the animals with dexamethasone.[20] In addition, glucocorticoid treatment did not inhibit neutrophil penetration via the endothelial cell junctions, although it was noted that the neutrophils subsequently remained between the endothelium and the basal membrane without reaching the perivascular tissue.[19,20] From these experiments it is clear that glucocorticoids affect neutrophil migration mainly by inhibiting an unidentified mechanism responsible for the transmigration through the basal membrane to the perivascular tissue.

The observation that dexamethasone does not affect MNCF-induced chemotaxis *in vivo* but does block the migration induced by IL-8 allows one to speculate about distinct mechanisms involved in the neutrophil migration induced by both cytokines. MNCF-induced chemotaxis *in vivo* must result from the protein's ability to promote the three stages of neutrophil emigration to tissues described above. IL-8-induced chemotaxis *in vivo*, however, may result from mechanisms involving a dexamethasone-sensitive release of chemotactic factors by resident cells such as mast cells[15] and/or the formation of a haptotactic gradient.[25,38] In relation to the generation of the haptotactic gradient, IL-8 and MNCF may recognize different ligands on the subendothelial matrix, and glucocorticoids may be able to block the expression of the IL-8 ligand while not affecting that of MNCF. The hypothesis of different ligands for IL-8 and MNCF is reinforced by the fact that MNCF, in contrast to IL-8 did not bind to heparin (Table 1), which is structurally related to heparan sulfate.[39] Heparan sulfate has been proposed to be the extracellular matrix ligand for IL-8 responsible for inducing neutrophil migration by the haptotactic mechanism.[39–41]

Based on the results of this study, we hypothesize that MNCF forms a dexamethasone-insensitive substrate bound gradient (haptotactic gradient) responsible for the last phase of neutrophil emigration through the basal membrane of acutely inflamed tissues. The previously reported dexamethasone blockade of the release of MNCF by macrophage monolayers stimulated by inflammatory stimuli[14] may at least in part explain why the last phase of neutrophil migration is impaired in dexamethasone pretreated animals.

In conclusion, a 54 kDa acidic protein, identified as macrophage-derived neutrophil chemotactic factor (MNCF), has lectin-like properties and causes neutrophil migration *in vivo* in animals treated with dexamethasone, as well as neutrophil chemotaxis *in vitro* by a haptotactic mechanism. MNCF is a candidate for a new cytokine and may play an important role in the transmigration of neutrophils to perivascular tissues.

### References

1. Springer TA. Traffic signals for lymphocyte recirculation and leukocyte emigration: the multistep paradigm. *Cell* 1994; **76:** 301–314.
2. Nathan CF. Secretory products of macrophages. *J Clin Invest* 1987; **79:** 319–326.
3. Takemura R, Werb Z. Secretory products of macrophages and their physiological functions. *Am J Physiol* 1984; **246:** C1-C9.
4. Werb Z, Banda MJ, Takemura R, Gordon S. Secreted proteins of resting and activated macrophages. In: Weir A, ed. *Handbook of Experimental Immunology*, Oxford: Blackwell Scientific Publications, 1986; 367–397.
5. Adams DO, Hamilton JA. Macrophages as destructive cells in host defense. In: Gallin JI, Goldstein IM, Snyderman R, eds. *Inflammation: Basic Principles and Clinical Correlates*, New York: Raven Press, 1992; 637–662.
6. Snyderman R, Phillips JK, Mergenhagen SE. Biological activity of complement *in vivo*. Role of C5 in the accumulation of polymorphonuclear leucocytes in inflammatory exudates. *J Exp Med* 1971; **134:** 1131–1143.
7. Ford-Hutchinson AW, Bray MA, Doig MV, Shipley ME, Smith JJH. Leukotriene $B_4$, a potent chemokinetic and aggregating substance released from polymorphonuclear leukocytes. *Nature* 1980; **280:** 264–265.
8. Palmblad J, Maimsten CL, Uden AM, Radmark O, Engstedt L, Samuelson B. Leukotriene $B_4$ is a potent and stereospecific stimulator of neutrophil chemotaxis and adherence. *Blood* 1981; **58:** 658–661.
9. Dustin ML, Rothlein R, Bhan AK, Dinarello CA, Springer TA. Induction by IL-1 and interferon-γ tissue distribution, biochemistry, and function of a natural adherence molecule (ICAM-1). *J Immunol* 1986; **137:** 245–254.
10. Rampart M, Williams TJ. Evidence that neutrophil accumulation induced by interleukin-1 requires both local protein biosynthesis and neutrophil CD18 antigen expression *in vitro*. *Br J Pharmacol* 1988; **94:** 1143–1148.
11. Faccioli LH, Souza GEP, Cunha FQ, Poole S, Ferreira SH. Recombinant interleukin-1 and tumour necrosis factor induce neutrophil migration *in vivo* by indirect mechanisms. *Agents Actions* 1990; **30:** 344–349.
12. Harmsen AG, Havel EA. Roles of tumour necrosis factor and macrophages in lipopolysaccharide-induced accumulation of neutrophils in cutaneous air pouch. *Infect Immun* 1990; **58:** 297–302.
13. Matsushima K, Morishita K, Yoshimura T, *et al.* Molecular cloning of a human monocyte-derived neutrophil chemotactic factor (MDNCF) and the induction of MDNCF mRNA by interleukin 1 and tumour necrosis factor. *J Exp Med* 1988; **167:** 1883–1893.
14. Cunha FQ, Ferreira SH. The release of a neutrophil chemotactic factor from peritoneal macrophages by endotoxin: inhibition of glucocorticoids. *Eur J Pharmacol* 1986; **129:** 65–76.
15. Ribeiro RA, Flores CA, Cunha FQ, Ferreira SH. IL-8 causes *in vitro* neutrophil migration by a cell-dependent mechanism. *Immunology* 1991; **73:** 472–477.
16. Souza GEP, Ferreira SH. Blockade by anti-macrophage serum of the migration of PMN neutrophils into the inflamed peritoneal cavity. *Agents Actions* 1985; **17:** 97–103.
17. Souza GEP, Cunha FQ, Melo R, Ferreira SH. Neutrophil migration induced by inflammation stimuli is reduced by macrophage depletion. *Agents Actions* 1988; **24:** 377–380.
18. Cronstein BN, Kimmel SC, Levin RI, Martiniuk F, Weissmann G. A mechanism for the antiinflammatory effects of corticosteroids: the glucocorticoid receptor regulates leucocyte adhesion to endothelial cells and expression of endothelial-leucocyte adhesion molecule 1 and intercellular adhesion molecule 1. *Proc Natl Acad Sci* 1992; **89:** 9991–9995.
19. Katori M, Oda T, Nagai K. A site of action for dexamethasone on leucocyte extravasation in the microcirculation. *Agents Actions* 1990; **29:** 24–26.
20. Oda T, Katori M. Inhibition site of dexamethasone on extravasation of polymorphonuclear leucocytes in the hamster cheek pouch microcirculation. *J Leucocyte Biol* 1992; **52:** 337–342.
21. Dias-Baruffi M, Roque-Barreira MC, Cunha FQ, Ferreira SH. Isolation and partial chemical characterization of macrophage-derived neutrophil chemotactic factor. *Med Inflamm* 1995; **4:** 257–262.
22. Dias-Baruffi M, Cunha FQ, Ferreira SH, Roque-Barreira MC. Macrophage-release of a neutrophil chemotactic factor (MNCF) induces neutrophil migration through lectin-like activity. *Agents Actions* 1993; **38:** C54–C56.
23. Edwards JCW, Sedgewick AD, Willoughby DA. The formation of structure with the features of synovial lining by subcutaneous injection of air. An *in vivo* tissue culture system. *J Pathol* 1981; **153:** 147–156.
24. Bignold LP. Kinetics of chemoattractant of polymorphonuclear leukocytes towards *N*-formyl peptide studied using a novel polycarbonate (Nuclepore) membrane in the Boyden Chamber. *Experientia* 1988; **44:** 518–521.
25. Rot A. Neutrophil attractant/activation protein-1 (interleukin 8) induces *in vitro* neutrophil migration by a haptotactic mechanism. *Eur J Immunol* 1993; **23:** 303–307.
26. Mello SBV, Farsk SHP, Sannomiya P, Garcia-Leme J. Inhibition of neutrophil chemotaxis and chemokinesis associated with a plasma protein in aging rats: selective depression of cell responses mediated by complement-derived chemoattractants. *J Leucocyte Biol* 1992; **51:** 46–52.
27. Santos-Oliveira R, Dias-Baruffi M, Thomaz SMO, Beltramini LM, Roque-Barreira MC. A neutrophil migration-inducing lectin from *Artocarpus integrifolia*. *J Immunol* 1994; **153:** 1798–1807.
28. Stein M, Keshav S. The versatility of macrophages. *Clin Exp Allergy* 1992; **22:** 19–27.
29. Lasky LA. Selectins: interpreters of cell-specific carbohydrate information during inflammation. *Science* 1992; **258:** 964–969.

30. Tozeren A, Ley K. How do selectins mediated leucocyte rolling in venules? *Biophys J* 1992; **63:** 700–709.
31. Muller WA, Weigel SA, Deng X, Philips DM. PECAM-1 is required for transendothelial migration of leukocytes. *J Exp Med* 1991; **178:** 449–460.
32. Vaporciyan AA, Delisser HM, Yan H, *et al.* Involvement of platelet-endothelial cell adhesion molecule-1 in neutrophil recruitment *in vivo. Science* 1993; **262:** 1580–1582.
33. Tanaka Y, Adams DH, Shaw S. Proteoglycans on endothelial cells present adhesion-inducing cytokines to leukocytes. *Immunol Today* 1993; **14:** 111–115.
34. Michel N, Whorton CM. Delay of the early inflammatory response by cortisone. *Proc Soc Exp Biol Med* 1951; **76:** 754–757.
35. Moon VH, Tershakovc GA. Influence of cortisone upon acute inflammation. *Proc Soc Exp Biol Med* 1953; **79:** 63–65.
36. Allison FJr, Smith JR, Wood WB. Studies on the pathogenesis of acute inflammation. II. The action of cortisone on the inflammatory response to thermal injury. *J Exp Med* 1955; **102:** 669–679.
37. Forsyth KD, Talbolt V. Role of glucocorticoids in neutrophil and endothelial adhesion molecule expression and function. *Mediators Inflammation* 1992; **1:** 101–106.
38. Rot A. Binding of neutrophil attractant/activation protein-1 (interleukin 8) to resident dermal cells. *Cytokine* 1992; **4:** 347–352.
39. Nader HB, Dietrich CP, Buonassisi V, Colburn P. Heparin sequences in the heparan sulfate chains of an endothelial cell proteoglycan. *Proc Natl Acad Sci* 1987; **84:** 3565–3569.
40. Webb LM, Ehrengruber MV, Clark-Lewis I, Baggiolini M, Rot A. Binding to heparan sulfate or heparin enhances neutrophil responses to interleukin 8. *Proc Natl Acad Sci* 1993; **90:** 7158–7162.
41. Huber AR, Kunkel SL, Todd III RF, Stephen JW. Regulation of transendothelial neutrophil migration by endogenous interleukin-8. *Science* 1991; **254:** 99–102.

ACKNOWLEDGEMENTS. This work was funded by the Fundação de Amparo à Pesquisa do Estado de São Paulo (FAPESP) and by the Conselho Nacional de Pesquisa e Desenvolvimento (CNPq), Brazil. The authors thank Mrs Sandra Maria Oliveira Thomaz, Maria Imaculada Bragheto, Neomésia Issajuara da Silva Freire and Sérgio Roberto Rosa for expert technical assistance.

**Received 31 March 1995; accepted 4 April 1995**
